# Metabolic syndrome in rheumatoid arthritis patients: Relationship among its clinical components

**DOI:** 10.1002/jcla.23666

**Published:** 2020-11-24

**Authors:** Mariel García‐Chagollán, Susana Elizabeth Hernández‐Martínez, Alma Elizabeth Rojas‐Romero, José Francisco Muñoz‐Valle, Ramón Sigala‐Arellano, Sergio Cerpa‐Cruz, José Javier Morales‐Núñez, José Alvaro Lomelí‐Nieto, Gabriela Macedo Ojeda, Jorge Hernández‐Bello

**Affiliations:** ^1^ Institute of Research in Biomedical Sciences University Center of Health Sciences (CUCS) University of Guadalajara Guadalajara México; ^2^ University Center for Exact Sciences and Engineering (CUCEI) University of Guadalajara Guadalajara México; ^3^ Clinical Pathology Laboratories Civil Hospital of Guadalajara Guadalajara México; ^4^ Rheumatology Service O.P.D. Civil Hospital of Guadalajara “Fray Antonio Alcalde” Guadalajara México; ^5^ Department of Public Health University Center of Health Sciences (CUCS) University of Guadalajara (UdG) Guadalajara Mexico

**Keywords:** Castelli's index, disease activity, lipid profiles, metabolic syndrome, rheumatoid arthritis, rheumatoid factor

## Abstract

**Background:**

Metabolic syndrome (MetS) prevalence in rheumatoid arthritis (RA) patients is known to vary considerably across the world. This study aimed to determine the prevalence of MetS in RA patients from western Mexico and to analyze the interrelation of the MetS components with the clinical variables of RA.

**Methods:**

This case‐control study included 216 RA patients and 260 control subjects (CS). MetS prevalence was determined according to the NCEP/ATP III and the Latin American Consensus of the Latin American Diabetes Association (ALAD) criteria.

**Results:**

MetS was observed in 30.6% RA patients and 33.3% of controls (*p* > 0.05) according to NCEP/ATP III and 28.7% in RA patients and 31.1% for controls using ALAD criteria. Total cholesterol, LDL‐C, and Castelli's I‐II indexes were lower in RA (*p* < 0.001) than in CS. The RA patients with MetS had more swollen joints than those without MetS (*p* = 0.018). In RA patients with MetS, DAS‐28 score correlated with smoking index (rho = 0.4601, *p* = 0.0004) and VLDL‐C (rho = 0.3108, *p* = 0.0056); similarly, rheumatoid factor (RF) correlated with age (rho = 0.2031, *p* = 0.0027), smoking index (rho = 0.3404, *p* < 0.0001), triglycerides (rho = 0.1958, *p* = 0.0039), and VLDL‐C (rho = 0.1761, *p* = 0.0162).

**Conclusions:**

The MetS prevalence in RA patients from western Mexico is not higher than controls; however, in RA patients with MetS, some inflammatory markers are associated with MetS components; thus, the control of MetS in RA could be beneficial to regulate disease activity.

## Graphical abstract

Relationship between rheumatoid arthritis (RA) and metabolic syndrome (MetS). The figure shows how the inflammatory markers of RA (IL‐6, TNF‐alpha, rheumatoid factor, etc,) can favor a response in different organs and tissues associated with the pathogenesis of MetS, such as the liver, pancreas, and the cardiovascular system, thus favoring the development of insulin resistance, atherosclerosis, and chronic inflammation. Likewise, the components of the Mets can favor the perpetuation of inflammation in RA.

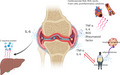

## 1 INTRODUCTION

Rheumatoid arthritis (RA) is an autoimmune inflammatory rheumatic disease associated with high levels of inflammatory markers and chronic comorbidities such as cardiovascular disease (CVD).^1^, ^2^, ^3^, ^4^ It has been demonstrated that RA life expectancy is reduced compared to that of the general population. Currently, CVD is the leading cause of mortality in patients with RA, being approximately 50% of the total of RA‐associated deaths.^1^, ^3^, ^5^

The RA is also associated with insulin resistance, dyslipidemia, and altered adipokines profile, which are included as metabolic syndrome (MetS) components.^6^ MetS is defined as a set of manifestations that are contemplated as cardiovascular risk factors (obesity, glucose intolerance, dyslipidemia, and hypertension), that along with systemic inflammation, contributes to CVD,^7^, ^8^, ^9^ and the prevalence of MetS has been associated with disease activity in RA.^8^, ^9^

In several populations, the overall risk of developing MetS seems to be significantly higher among patients with RA than in healthy controls; however, this differs considerably, based upon the diagnostic criteria used and the population ancestry.^10^ On the other hand, the relationship between RA and MetS showed a significantly negative correlation in other countries such as Korea.^11^ In Latin American countries, including Mexico,^12^, ^13^, ^14^ there have been few solid studies regarding this topic^10^; therefore, more research is needed to understand the metabolic changes of MetS in RA and its critical role in the development of CVD in RA patients from different geographic regions.

The biochemical profiles in RA occasionally does not reflect what observed in the general population, for example, low total cholesterol levels in RA patients have been associated with an increased cardiovascular risk, as well as, a high body mass index (BMI) has a protective effect on the amount of joint destruction in small joints in early RA.^15^, ^16^, ^17^

The RA association with MetS also differs between chronic and early RA,^3^, ^9^ and the role of different characteristics of the disease, such as disease duration, activity, and the frequency of treatments, is not well defined yet.^17^, ^18^, ^19^, ^20^

Identifying MetS components in RA patients could provide a crucial opportunity for a preventive intervention; however, the controversy is evident about which factors are the most important to drive RA‐associated MetS.^18^, ^19^, ^20^ This study aimed to assess the prevalence of MetS in RA patients from western Mexico and analyze the interrelation of the MetS components with the clinical variables of RA.

## 2 MATERIALS AND METHOD

### 2.1 Subjects

From February 2017 to May 2019, 216 RA patients and 270 control subjects (CS) were enrolled. The sample size was calculated with OPENEPI calculator^21^ to detect an 80% statistical power with a confidence degree of 95% and an expected prevalence of MetS in RA patients of 17.5% reported in a previous study.^12^ The Ethics Committee approved the protocol of the Hospital “Fray Antonio Alcalde” (HCG/CEI‐0153/18); written informed consent was obtained from all individuals.

According to the ACR 1987 classification criteria,^22^RA patients were diagnosed by a rheumatologist. They were enrolled in the study when they came to their clinical control visit at the Department of Internal Medicine/Rheumatology of the OPD Hospital Civil de Guadalajara "Fray Antonio Alcalde" in the state of Jalisco, Mexico. The rheumatologist conducted a medical record and 28 joints evaluation to estimate the clinical activity using the Disease Activity Score‐28 (DAS‐28).^23^

The CS group included subjects without either known medical condition or treatment, with similar age, sex, and geographic regions as the RA group. They were randomly selected from the clinical laboratory of the OPD Hospital Civil de Guadalajara "Fray Antonio Alcalde" when they went for a regular health check‐up. The evaluation of physical activity, food consumption, and smoking index (number of cigarettes smoked per day × years of tobacco use) was realized during a personal interview in both study groups. Subjects were excluded from the study if they suffered from congestive heart disease, renal disease, endocrinological abnormalities, or were under medications that altered blood pressure, glucose, or lipid metabolism.

### 2.2 Anthropometric measurements

Anthropometric measurements were determined for all individuals with absolute reliability. Weight was measured with the subjects wearing lightweight clothes without shoes, and after an overnight fasting, using a standard scale (TANITA BC‐568 INNERSCAN). Height was measured and approximate to the nearest 0.1 cm using a Seca 213 mobile stadiometer, with the participant standing in a vertical plane with head in the Frankfort horizontal plane. Waist circumference was measured at the minimum circumference between the iliac crest and the lowest rib. Blood pressure was measured twice, while the patients were seating and resting for 5 min, using a digital sphygmomanometer. BMI was calculated as weight (kg) divided by height squared (m^2^).

### 2.3 Biochemical analysis

A blood sample was obtained from all individuals after an overnight fast (12 h). A full fasting lipid profile (triglycerides, total serum cholesterol, high‐density lipoprotein cholesterol [HDL‐C], low‐density lipoprotein cholesterol [LDL‐C]) and fasting blood glucose (FBS) concentrations were obtained using automated equipment (The Beckman Coulter AU5800 (Beckman Coulter Inc, Brea CA, USA). Serum C‐reactive protein (CRP) levels were determined by immunoturbidimetry (OSR6147). Castelli's Risk Index I was calculated as total serum cholesterol (mg/dl) divided by HDL‐C (mg/dl), and Castelli's Risk II was calculated as LDL‐C (mg/dl) divided by HDL‐C (mg/dl).

### 2.4 MetS definition

Subjects were diagnosed with MetS based on NCEP ATP III^24^ and The Latin American Consensus of the Latin American Diabetes Association (ALAD)^25^ criteria. Demographic data were collected by questionnaire.

### 2.5 Statistical analysis

Statistical analysis was performed using GraphPad Prism v6.0. The Shapiro‐Wilk normality test was applied to verify the normal distribution of the data. As it is appropriate, parametric or non‐parametric tests were used for the analysis. The nominal discontinuous variables were expressed as frequencies (number and percentages); the continuous variables with parametric distribution were expressed as means ± standard deviation (SD) and the non‐parametric variables as medians and interquartile ranges. The chi‐square (χ^2^) test was used to compare proportions. Student's t test was applied for two groups parametric quantitative analysis, and the Mann‐Whitney U test was used for non‐parametric quantitative determinations. Spearman's correlation analysis was used to discover the strength of a link between two sets of data. A multivariate logistic regression model was used to examine the independence of the predictors of RA parameters and MetS. A probability (*p*) value of less than 0.05 (*p* < 0.05) was considered significant.

## 3 RESULTS

### 3.1 Clinical and demographic characteristics

The demographic, clinical, and laboratory characteristics of RA patients and control subjects (CS) are described in Table 1. RA patients had lower weight and size than CS (*p* < 0.05). Also, RA patients reported less food intake per day compared to controls. No significant differences were observed between the two groups for age, sex, waist circumference, BMI, systolic blood pressure, physical activity, and smoking index. Most of the patients (75%) were diagnosed with RA 15 years before their inclusion in the study (median 7.8 years), and 50% had a DAS‐28 score < 3.1, which indicates a low activity disease. 70% of patients were treated with at least one disease‐modifying antirheumatic drug and 70% with nonsteroidal anti‐inflammatory drugs. Another minority of individuals was being prescribed <5 mg/day of prednisone (27%).

**Table 1:** Sociodemographic, anthropometric, and clinical parameters in CS and RA patients

	CS (n = 270)	RA (n = 216)	*p*‐value
Demographics and anthropometric components			
Sex (Female/Male)	(240/30)	(194/22)	0.769
Age (years)^†^	44 (33–55)	46 (37−55)	0.074
Weight (kg)^†^	69 (62−81)	65 (55−73)	**0.0001**
Height (cm)^†^	1.61 (1.56−1.65)	1.56 (1.52−1.62)	**<0.0001**
Waist circumference (cm)^†^	98 (88−105)	96 (87−103)	0.0877
BMI (kg/m^2^) ^‡^	27 (23−30)	26 (23−30)	0.0703
Systolic pressure (mmHg)^†^	116 (105−125)	120 (108−126)	0.7302
Diastolic pressure (mmHg)^†^	80 (70−82)	78 (80−82)	0.4601
Meals per day^‡^	3.53 ± 1.236	2.91 ± 0.70	**<0.0001**
Physical activity n (%)			
Never	100 (37)	68 (32)	0.343
Occasionally	59 (22)	57 (26)	
Frequently	111 (41)	91 (42)	
Smoking index ^‡^	1.46 ± 4.6	1.8 ± 4.6	0.7580
Characteristics of RA			
DAS‐28 score^†^		3.1 (2.1−4.8)	−
Evolution of RA (years)^†^	−	7.82 (2−15)	−
Rheumatoid factor (UI/ml)^†^		137 (20‐712)	−
Drug treatment n (%)			
NSAIDs	−	189 (70)	−
Prednisone	−	58 (27)	−
DMARDs			
Sulfasalazine	−	73 (34)	−
Chloroquine	−	60 (28)	−
Methotrexate	−	189 (70)	−

Note

According to the normal distribution, results expressed as median and interquartile ranges, Mann‐Whitney test ^†^ or mean ± standard deviation, Student's t test^‡^. Bold letters indicate statistically significant results.

Abbreviations: CS, control subject; DAS‐28, disease activity score 28; DMARDs, disease‐modifying antirheumatic drugs; NSAIDs, nonsteroidal anti‐inflammatory drugs; RA, rheumatoid arthritis.

### 3.2 Biochemical and paraclinical parameters of CS and RA patients

Glucose, lipid profile, and the Castelli's risk index‐I (CRI‐I) and (CRI‐II) were determined in both study groups. Table 2 shows that the levels of glucose, HDL‐C, triglycerides, and VLDL‐C were similar (*p* > 0.05) between CS and RA patients. The total serum cholesterol and LDL‐C were higher in CS than in RA patients (*p* = 0.0045 and *p* = 0.0006, respectively); similarly, CRI‐I (CT/ HDL‐C: CS 4.27 [3.5–5] vs. RA 3.89 [3.2–4.5], *p* = 0.0002) and CRI‐II (LDL‐C/HDL‐C: CS 2.65 [2.1–3–3] vs. RA 2.3 [1.8–2.8], *p* < 0.0001) were higher in CS than in RA patients.

**Table 2:** Biochemical and Paraclinical Parameters of Patients with RA and CS

	CS (n = 270) Median (P25−P75)	RA (n = 216) Median (P25−P75)	*p*‐value
Glucose (mg/dl)	93 (88−101)	95 (89−100)	0.2539
Triglycerides (mg/dl)	109 (78−164)	99 (78−135)	0.1434
Total cholesterol (mg/dl)	183 (150−213)	168 (143−196)	**0.0045**
HDL‐C (mg/dl)	42 (35−48)	43 (37−53)	0.0681
LDL‐C (mg/dl)	116 (90−134)	102 (82−121)	**0.0006**
VLDL‐C (mg/dl)	22 (16−33)	20 (16−27)	0.2975
CRI‐I	4.27 (3.5−5)	3.89 (3.2−4.5)	**0.0002**
CRI‐II	2.65 (2.1−3.3)	2.3 (1.8–2.8)	**<0.0001**

Note

Data expressed as median and interquartile ranges (25th percentile [P25] and 75th percentile [P75], Mann‐Whitney test. Bold letters indicate statistically significant results.

Abbreviations: CRI‐I, Castelli's Risk Index I; CRI‐II, Castelli's Risk Index II; HDL‐C, high‐density lipoprotein cholesterol; LDL‐C, low‐density lipoprotein cholesterol; milligrams (mg), deciliters (dl); VLDL‐C, very‐low‐density lipoprotein cholesterol.

### 3.3 Frequency of MetS in CS and RA patients

According to NCEP/ATP‐III criteria, the overall frequency of MetS was 33.3% in CS and 30.6% in RA patients (*p* = 0.514, Table 3). This result did not suffer significant variations after adjusting for height and weight (*p* = 0.325). The waist circumference criterion was more frequent (*p* = 0.0150) in patients with RA (68.5%) than in CS (57.7%). Based on ALAD criteria, 31.1% CS and 28.7% RA patients had MetS (*p* = 0.5218).

**Table 3:** Frequency of MetS according to who different criteria in CS and RA patients

	CS (n = 270)	RA (n = 216)	*p*‐value
	n (%)	n (%)	
NCEP/ATP‐III criteria			
Waist circumference > 102 cm in men and >88 cm in women	156 (57.7)	148 (68.5)	**0.0150**
Hypertriglyceridemia (TG ≥ 150 mg/dl)	75 (27.7)	42 (19.4)	0.6605
Hypertension (BP ≥ 130/85 mmHg)	54 (20)	41 (19)	0.7784
Hyperglycemia ≥ 110 mg/dl	63 (23.3)	52 (24)	0.8485
Low HDL‐C < 40 mg/dl in men and <50 mg/dl in women	163 (60.3)	136 (62.9)	0.5594
MetS	90 (33.3)	66 (30.6)	0.5145
ALAD criteria			
Waist circumference > 94 cm in men and > 88 cm in women	182 (67.4)	149 (68.9)	0.7114
MetS	84 (31.1)	62 (28.7)	0.5218

Note

Results expressed as number (n) and percentage (%). Bold letters indicate statistically significant results.

Abbreviations: ALAD, Latin American Diabetes Association; BP, blood pressure; NCEP/ATP‐III, The National Cholesterol Education Program (NCEP) Adult Treatment Panel III (ATP III).

### 3.4 Relationship of MetS parameters with RA characteristics

As it was expected, Table 4 shows that all lipid and paraclinical components of MetS were higher in RA patients with MetS (*p* < 0.05) than those without MetS. However, the inflammatory parameters (CRP, ESR, RF) and the activity of the disease (DAS‐28 index) did not show significant differences between both groups (*p* > 0.05). Multivariate logistic regression analysis did not show any variable with statistical significance (data not shown).

**Table 4:** Characteristics of RA patients based on the presence or the absence of MetS

	RA with metabolic syndrome (n = 60)	RA without metabolic syndrome (n = 156)	*p*‐value
Age (years)^‡^	53.23 ± 11.23	42.56 ± 12.34	**<0.0001**
Sex (Female/Male)	8/52 (13.3/76.7)	14/142 (8.9/91.1)	0.3420
BMI (kg/m^2^)^‡^	28.54 ± 3.727	25.59 ± 5.229	**<0.0001**
Weight (kg)^‡^	73.51 ± 12.22	62.42 ± 13.20	**<0.0001**
Waist circumference (cm)^†^	101 (98–107.5)	90.25 (83.50–100)	**<0.0001**
Systolic pressure (mmHg)^†^	129.5 (120–138)	114 (105–122)	**<0.0001**
Diastolic pressure (mmHg)^†^	83 (78–86)	75 (70–80)	**<0.0001**
Evolution of RA (years)^‡^	7.75 ± 7.90	7.89 ± 7.43	0.7274
DAS‐28^†^	3 (2.1–5.1)	3.15 (2–4)	0.6890
Swollen joints^†^	1 (0–4)	0 (0–1)	**0.0183**
Painful joints^†^	3 (0–6)	2 (0–6)	0.6114
ESR (mm/h)^†^	25 (16–34)	25 (18–35)	0.8206
CRP (mg/dl)^†^	6.05 (3.7–9.3)	5.950 (2.7–12.3)	0.6599
RF (UI/ml)^†^	107.2 (40.4–147.4)	86.2 (20.2–145.3)	0.3157
Glucose (mg/dl)^‡^	104.4 ± 13.6	92.97 ± 7.613	**<0.0001**
Total Cholesterol (mg/dl)^†^	211 (189.5–271)	161 (140–188)	**<0.0001**
Triglycerides (mg/dl)^†^	147 (99–174)	94 (76–124)	**<0.0001**
HDL‐C (mg/dl)^†^	39 (34–48)	45 (39–55)	**0.0010**
LDL‐C (mg/dl)^†^	119 (97–130)	92 (98–117)	**<0.0001**
VLDL‐C (mg/dl)^†^	29 (20–38)	19 (15–25)	**<0.0001**

Note

According to the normal distribution, results expressed as median and interquartile ranges, Mann‐Whitney test^†^ or mean ± standard deviation, Student's t test^‡^. Bold letters indicate statistically significant results.

Abbreviations: BMI, Body mass index; CRP, C‐reactive protein; ESR, erythrocyte sedimentation rate; HDL‐C, high‐density lipoprotein cholesterol; LDL‐C, low‐density lipoprotein cholesterol; milligrams (mg), deciliters (dl); VLDL‐C, very‐low‐density lipoprotein cholesterol.

A Spearman correlation analysis was performed between the activity variables of RA (RF, CRP, ESR, and disease activity) and the parameters of MetS. Table 5 shows positive correlation of DAS‐28 score with the smoking index (rho = 0.4601, *p* = 0.0004) and VLDL‐C (rho = 0.3108, *p* = 0.0056) levels; similarly, RF correlated with age (rho = 0.2031, *p* = 0.0027), smoking index (rho = 0.3404, *p* < 0.0001), triglycerides (rho = 0.1958, *p* = 0.0039), and VLDL‐C (rho = 0.1761, *p* = 0.0162). Monotherapy versus multi‐therapy in the treatment of RA patients showed no association with any biochemical variables (*p* > 0.05, data not shown).

**Table 5:** Correlation between RF, CRP, ESR, and DAS‐28 with anthropometric and biochemical components of MetS in RA patients with MS

	DAS‐28‐score		ESR (mm/hr)		RF		CRP (mg/L)	
	rho	*p*‐value	rho	*p*‐value	rho	*p*‐value	rho	*p*‐value
Age	0.092	0.370	0.013	0.842	**0.203**	**0.002**	−0.044	0.512
Smoking index	**0.460**	**<0.0001**	0.107	0.163	**0.340**	**<0.0001**	0.084	0.278
Weight	−0.167	0.102	−0.111	0.101	−0.028	0.675	**0.176**	**0.009**
Waist circumference	−0.162	0.113	0.044	0.520	0.040	0.551	**0.198**	**0.003**
BMI	−0.162	0.113	−0.108	0.112	−0.030	0.654	**0.173**	**0.010**
Triglycerides	0.179	0.080	0.114	0.093	**0.195**	**0.003**	0.088	0.193
Cholesterol	−0.028	0.781	0.121	0.073	0.028	0.680	−0.046	0.497
HDL‐C	−0.154	0.131	0.004	0.945	0.047	0.487	−0.002	0.973
Glucose	0.069	0.501	−0.104	0.126	0.034	0.618	0.049	0.472
Systolic pressure	0.024	0.811	−0.140	0.309	0.020	0.770	0.067	0.322
Diastolic pressure	−0.056	0.585	−0.067	0.323	0.020	0.770	0.128	0.060
LDL‐C	−0.113	0.270	0.124	0.067	−0.050	0.464	−0.050	0.461
VLDL‐C	**0.310**	**0.005**	0.103	0.158	**0.176**	**0.016**	0.046	0.527
CRI‐I	0.088	0.393	0.129	0.057	0.0001	0.999	−0.029	0.668
CRI‐II	−0.060	0.560	0.112	0.098	−0.065	0.338	−0.059	0.386

Note

Data analyzed by Spearman's rank‐order correlation (rho). Bold letters indicate statistically significant results.

Abbreviations: BMI, Body mass index; CRI‐I, Castelli's Risk Index I; CRI‐II, Castelli's Risk Index II; CRP, C‐reactive protein; ESR, erythrocyte sedimentation rate; HDL‐C, high‐density lipoprotein cholesterol; LDL‐C, low‐density lipoprotein cholesterol; milligrams (mg), deciliters (dl); VLDL‐C, very‐low‐density lipoprotein cholesterol.

## 4 DISCUSSION

Ethnical, nutritional, and sociodemographic status are some factors determining the prevalence of MetS.^10^ In RA, insulin resistance may be produced secondary to the inflammatory component of the disease, and it may also increase the risk of MetS and cardiovascular complications.^26^ Thus, understanding the pathophysiology underlying this syndrome may help us explain these risk factors and their prevalence in this type of patient.^27^

Research about MetS prevalence in RA patients has shown a wide range of estimates globally; therefore, more research is needed to understand the interrelationship between MetS and RA in different geographic regions. A recent meta‐analysis evaluated the prevalence and risk of MetS in RA patients, and it showed a general prevalence of MetS of 30.65%, but the results varied from 14.32% to 37.83%, based upon the diagnostic criteria used. The overall pooled odds ratio of MetS in RA patients was of 1.44 compared to controls, but this ranged from 0.70 to 4.09, depending on the criteria used and ethnicity.^10^

In healthy Mexicans, there has been reported a high prevalence of MetS among adults, in comparison with reports from other countries, including the United States and Latin America; the estimated Prevalence of MetS according to the ATP III and WHO criteria is 36% and 31%, respectively,^28^ similar to that observed in this study (ATP‐III criteria = 33.3%, and ALAD criteria = 31.1%). There are also a few studies regarding MetS in RA patients in the Mexican population from northern, southern, and central,^12^, ^13^, ^14^ but not from western Mexico.

Although many studies have reported a higher prevalence of MetS among RA patients than controls,^8^, ^10^ others performed in central Mexico, Argentina, and Iran have reported otherwise,^12^, ^19^, ^29^ being the reason of why the results are controversial. In the present work, no differences were observed in the MetS prevalence among RA and CS (*p* > 0.05), but there was a tendency of the low prevalence of MetS in RA patients in comparison with CS according to NCEP/ATP‐III and ALAD criteria, similar to that reported in the population of central Mexico (RA = 26.3% vs CS = 30%, *p* < 0.01) (RA = 26.3% vs CS = 30%, *p* < 0.01).^12^ In Iranian^30^ and Greek populations,^31^ similar to us, these differences have not been observed either. In the Iran Population, the only marked difference observed was the significantly longer duration of the disease in RA patients with MetS, compared to those without MetS.^30^

Discrepancies regarding the higher or lower frequency of MetS in RA in comparison with CS could be due to age, sex, ancestry, treatment, and geographic residency. In this study, no differences in the presence of these variables were identified between both study groups. Nevertheless, a potential weakness of this study could be the differences observed between weight, size, and meals per day between groups (*p* < 0.001), being RA patients lighter and less tall than controls, but not BMI changes were observed. RA patients also reported a lower number of meals per day, even though multivariate analysis models did not show any changes according to these variables (data not shown).

Considering the previous findings among the Mexican population,^12^, ^13^, ^14^ we suggest that ethnicity could be a significant component of the differences between MetS prevalence in RA patients from Mexico and other countries. However, as nutritional habits are major risk factors for MetS, a more detailed and validated survey evaluation of food consumption is also further required to clear this hypothesis, including the amount of food, energy, and nutrients consumed, or patterns of food consumption and physical activity.

The differences regarding weight (*p* = 0.0001) and the number of meals eaten per day (*p* < 0.0001) between RA and CS could be due to a "rheumatoid cachexia" status in RA patients, which includes a loss of cell mass (skeletal muscle) and the increase in fat mass, resulting in an apparently stable body composition throughout life.^32^ It has been studied that the development of depressive factors due to RA pathology's disabling effects^33^ could be one of the likely causes answers to minor food intake throughout the day, and also to the low weight among RA patients.

An interesting finding involves the low total cholesterol and LDL‐C levels and Castelli's I‐II atherogenic indices in RA patients compared to CS (*p* < 0.001), which indicates lower cardiovascular risk in these patients. In a previous study performed in the Mexican population, the average of both indices in patients with RA was 4.36 and 2.59, similar to the present study; authors attribute this finding to the treatment with hydroxychloroquine, since patients treated with this drug had a lower frequency of atherogenic dyslipidemia.^34^ Also, methotrexate seems to exert other cardioprotective properties on lipids and endothelium in RA patients, as serum of patients treated with methotrexate showed an increased cholesterol efflux capacity mediated by ABCG‐1 and scavenger receptor class B type I. In vitro, methotrexate inhibits foam cell formation by promoting reverse cholesterol transport through activation of adenosine A2 receptor, and it increases in cholesterol 27‐hydroxylase and ABCA‐1.^35^, ^36^ In our study, only 70% of patients were taking pharmacological therapy combined, including methotrexate and chloroquine, so we were unable to identify the effect of drugs on lipid profiles; therefore, it is pertinent that future studies may contemplate this variable to clarify the relationship that is projected to lower atherogenic risk in patients with RA.

Controversially, there are also reports showing decreases in total cholesterol and LDL‐C prior and during RA development, but even so, the risk of CVD is latent in these patients^37^; this paradoxical effect is not well understood yet, and its interactions with inflammation and CVD in RA are known to be complex.^38^

It is not clear whether the components of MetS precede the appearance of inflammatory diseases or if they are a consequence of its complications. Chung et al (2008) reported that the frequency of MetS is higher in patients with chronic RA (42%) than those with early RA (30%).^39^ In this study, the RA evolution time was not concluded to be a determining factor for MetS, as RA patients with MetS had a similar time of evolution than those without it. However, we must consider that RA patients with MetS were older than those without MetS (years, 53.23 vs 42.56, respectively, *p* < 0.0001), which is a well‐understood risk factor for MetS.^40^

The inflammatory markers or disease activity of RA did not show any significant differences according to the presence or absence of MetS, except for the number of swollen joints, as these were more prevalent in the patients with MetS (Median [IQR] = RA with MetS 1 (0–4) vs RA without MetS 0 (0–1), *p* = 0.018). This association could be due to the overweight in the MetS groups since it has been reported a positive correlation between BMI and the number of swelling joints in RA patients.^41^ Based on these findings, we suggest that MetS is not a subsequent RA pathophysiology event, but it could be a promoter of some of components that influence the disease activity or disability.

On the other hand, there was a positive correlation between disease activity (DAS‐28 score) in RA patients with MetS with smoking index and VLDL‐C levels. This finding could be explained by the effect of tobacco smoking on tissue protein citrullination, and also detonate of autoantibodies synthesis, which show to induce the disease progression and disease activity in patients with rheumatoid arthritis.^42^, ^43^, ^44^ On the other hand, the association of DAS‐28 and VLDL‐C levels is not clear yet, but it has been reported that RA patients with high disease activity had alterations of the lipid profile.^38^, ^45^

Rheumatoid factor (RF) correlated with age and smoking index, which agrees with other reports^46^, ^47^, ^48^; likewise, RF correlated with triglycerides, which could be explained by this autoantibody´s positive correlation with a high RA disease activity,^49^ a status associated with lipids alteration.

Regarding the correlation of CRP with some MetS components such as weight, waist circumference, and BMI, it could be explained by the fact that because these factors are the primary determinant of chronic inflammation in subjects with the MetS and are strongly related with proinflammatory cytokines such as IL‐6, which induces the synthesis of CRP.^50^

Some limitations of this study are that most patients were being treated on a combined drug therapy, since some drugs like chloroquine could alter metabolism, lipid profile, and inflammation markers in RA. Also, patients with a low disease activity (DAS‐28 ranges of 2.6–3.9) were overrepresented because most of the patients (75%) had DAS‐28 < 3.9.

In conclusion, the present study's findings suggest that there is no sufficient evidence of a higher prevalence of metabolic syndrome in RA patients from western Mexico compared with controls. RA patients' age and female sex were associated with MetS, just as reported in the general population, implicating that MetS could be an isolated event of RA course in our population. However, in RA patients with MetS, CRP, RF levels, and disease activity score (DAS‐28) are associated with MetS components, so in this clinical context, the control of MetS in RA could effectively control disease activity outcomes. Further research around the topic might explore the basis of the lower lipid profiles and Castelli's indexes in RA patients. Also, the control study should have a better handle of the pharmacologic effects on RA patient´s therapy to establish the pathophysiology of lipid abnormalities in RA.

## CONFLICT OF INTEREST

There is no conflict of interest in this work.

## Data Availability

All the data related to this work are available at the corresponding author. The data used to support the findings of this study are included in the article.
